# Understanding Lignin-Degrading Reactions of Ligninolytic Enzymes: Binding Affinity and Interactional Profile

**DOI:** 10.1371/journal.pone.0025647

**Published:** 2011-09-29

**Authors:** Ming Chen, Guangming Zeng, Zhongyang Tan, Min Jiang, Hui Li, Lifeng Liu, Yi Zhu, Zhen Yu, Zhen Wei, Yuanyuan Liu, Gengxin Xie

**Affiliations:** 1 College of Environmental Science and Engineering, Hunan University, Changsha, China; 2 Key Laboratory of Environmental Biology and Pollution Control, Hunan University, Ministry of Education, Changsha, China; 3 State Key Laboratory for Chemo/Biosensing and Chemometrics, College of Biology, Hunan University, Changsha, China; Russian Academy of Sciences, Institute for Biological Instrumentation, Russian Federation

## Abstract

Previous works have demonstrated that ligninolytic enzymes mediated effective degradation of lignin wastes. The degrading ability greatly relied on the interactions of ligninolytic enzymes with lignin. Ligninolytic enzymes mainly contain laccase (Lac), lignin peroxidase (LiP) and manganese peroxidase (MnP). In the present study, the binding modes of lignin to Lac, LiP and MnP were systematically determined, respectively. Robustness of these modes was further verified by molecular dynamics (MD) simulations. Residues GLU460, PRO346 and SER113 in Lac, residues ARG43, ALA180 and ASP183 in LiP and residues ARG42, HIS173 and ARG177 in MnP were most crucial in binding of lignin, respectively. Interactional analyses showed hydrophobic contacts were most abundant, playing an important role in the determination of substrate specificity. This information is an important contribution to the details of enzyme-catalyzed reactions in the process of lignin biodegradation, which can be used as references for designing enzyme mutants with a better lignin-degrading activity.

## Introduction

Lignin, a very complex biopolymer in the plant cell wall, is usually treated as contaminant in agriculture and in the pulp/paper industry [1]–[3]. Its degradation is important for carbon recycling of the biosphere [4], [5]. Large numbers of accumulating lignin could cause serious environmental problems [2]. However, lignin is dramatically resistant towards chemical degradation [1]. Fortunately, various microorganisms can produce a battery of enzymes to degrade lignin [3]. Much attention has been drawn to the development of environmentally friendly technologies for treating lignin by ligninolytic enzymes. The enzymes involved in lignin decay mainly include Lac, LiP and MnP [3]. Among the process of lignin biodegradation, lignin first interacts with ligninolytic enzymes and further its conformation is changed to achieve an overall best-fit, giving rise to the formation of radicals and the breakdown of various bonds in lignin [2], [6], [7]. Lac, a polyphenol oxidase, has been found for many years in fungi [3]. Lac alone can only oxidize phenolic lignin units, but is also capable of degrading non-phenolic lignin units in the presence of synthetic mediators [7]. LiP and MnP consisting of heme-containing glycoproteins were first discovered in *Phanerochaete chrysosporium* (*P. chrysosporium*) [6]. LiP catalyzes the oxidation of lignin by electron transfer, non-catalytic cleavages of various bonds and aromatic ring opening [6]. MnP is an extracellular heme enzyme with manganese as a cofactor [2]. Mn^II^ interacts with MnP using H_2_O_2_ as oxidant, leading to the formation of Mn^III^-oxalate complex which is able to oxidize the substrate lignin [2], [8]. A lot of work has been done to explore the activities of ligninolytic enzymes. An amperometric enzyme sensor has been developed by our group to detect simultaneously the activities of LiP and MnP [9]. Our previous work has shown inoculation times had a positive or negative effect on the activities of ligninolytic enzymes [4]. The biochemistry of LiP and MnP has been well studied, and their encoded genes have been also identified [10]. Martinez and co-workers located these genes in *P. chrysosporium* genomes with bioinformatics method [11].

The ability of LiP, MnP and Lac to degrade lignin has been studied in agriculture waste composting and in diverse industrial processes including pulp delignification, and bioremediation of soils and water, but this ability is non-identical between these three types of enzymes [3]. This may be due to that enzyme-substrate interactions are different. The study of the interactive mechanisms involved in enzymes and lignin is indeed important in understanding enzyme reactions and contributing to the improvement of the pulping and bleaching technologies [12], [13]. Monitoring the interactions of lignin with ligninolytic enzymes may provide further insights into the development of the lignin biodegradation technologies. Early experimental results suggested that ligninolytic enzymes were capable of degrading lignin by direct interactions of ligninolytic enzymes with lignin in terms of a long-range electron transfer process [12]–[14]. However, little is known about the effect of ligninolytic enzymes' structures on the lignin biodegradation at the molecular level. Ligninolytic enzyme-lignin interactions can be revealed by experimental techniques, but atomic details of interaction cannot be given [15]. Moreover, experimental techniques to investigate the interaction mechanisms are time-consuming and expensive. Bioinformatics methods have been used to analyze simple sequence repeats in pre-microRNAs of environmental microorganisms [16]. Park et al performed a combined approach of the experiments and molecular docking to study interaction mechanisms between alkyl phenol and *Coprinus cinereus* peroxidase (CIP) [17]. Molecular docking is a method that predicts the binding mode of a ligand to a receptor, and has been extensively used in rational design of drug [18], [19]. In general, the docking conformations need to be examined by MD simulations [15], [20]. Aristilde et al employed Monte Carlo molecular simulation to elucidate the binding modes of oxytetracycline with a smectite clay [21]. Thus, in order to propose a plausible binding conformation between ligninolytic enzymes and lignin which might explain the observed experimental oxidation activity of the ligninolytic enzymes during agricultural waste composting and in the pulp/paper industry, we carried out automatic molecular docking simulations using the Molegro Virtual Docker (MVD) software. The dynamic stability of ligninolytic enzyme-lignin binding modes was further analyzed using MD simulations. Information from this study can be used to design promising Lac, LiP or MnP mutants with a better oxidation activity toward lignin.

## Results and Discussion

Due to potential value of Lac, LiP and MnP to the development of economy and environmental protection, their catalytic mechanisms in relation to lignin biodegradation have been well revealed for many years [3]. The best-studied lignin-degrading organism is *P. chrysosporium* [2]. Thus, two of three crystal structures in this study are from *P. chrysosporium*. Exception includes Lac (PDB code 1GYC) from *Trametes versicolor* [7]. Despite the effective use of *P. chrysosporium* and other fungi for degrading lignin waste, this method is limited to some extent by poor knowledge about the interaction mechanisms between ligninolytic enzymes and lignin. To address this problem, we performed molecular docking and MD simulation using available structural information. Molecular docking and molecular simulation have been introduced to tackle the environment problems including oxidative polymerization of alkyl phenols [17] and adsorption of antibiotic contaminants [21], respectively, but a combined approach of these two methods is not used. In particular, in these studies, some structures are not available in databases, and thus have to be determined by X-ray diffraction (XRD), solid-state nuclear magnetic resonance (NMR) spectroscopies and other experiments [17], [21]. As opposed to these studies, all structures including ligand and receptor in the present study are available in databases, and hence no experiments are required. The detailed information about selected Lac, LiP, and MnP structures is listed in Table 1. Our docking results revealed that the lignin selected in this study had strong binding affinities with the Lac, LiP and MnP. Their docked conformations indicated similarities and differences. These similarities might lay a common foundation for the lignin-degrading activity of analyzed ligninolytic enzymes, whereas these differences were likely to partly lead to their non-identical lignin-degrading ability. The docking experiments carried out gave good structural insights into how various ligninolytic enzymes interacted with lignin in acting as catalysts. Our results have important contribution to the details of enzyme-catalyzed reactions in the process of lignin biodegradation, which can broaden our knowledge of lignin-biodegrading techniques.

**Table 1:** Overview of ligninolytic enzyme PDB codes, resolutions (R), molecular weight (MW, g/mol), number of bonds (NB), number of residues (NR), and MolDock score and Re-Rank score of the best docking poses for lignin ligand into the biding pockets of ligninolytic enzymes.

Ligninolytic enzyme	PDB code	R (Å)	MW	NB	NR	Pocket volume (Å^3^)	Enzyme-lignin complex^a^	MolDock score (kcal mol^−1^)	Re-Rank score (kcal mol^−1^)
Lac	1GYC	1.90	55989.82	7581	499	69.63	Lac-lignin	-127.77	-103.76
LiP	1LLP	1.70	37969.45	5058	343	250.88	LiP-lignin	-156.03	-123.90
MnP	3M5Q	0.93	38931.55	5225	357	211.46	MnP-lignin	-142.33	-128.40

a Refers to the best docking complex.

MolDock score and Re-Rank score refer to two score functions of MVD, and reflect the binding energy of system. For more detailed information, please see Results and Discussion section.

### Binding affinity

It has been confirmed that the lignin-degrading mechanisms of Lac and LiP are associated with direct interactions between lignin and them [13], [14], but detailed binding orientations and interaction profiles between ligninolytic enzymes and lignin were not reported until now. Thus, docking was done to position lignin into the active sites of ligninolytic enzymes and to determine the possible binding affinity using MVD which is very robust due to its effective scoring functions [22]. The docking accuracy of MVD has been well evaluated in various experiments by root-mean-square derivation (RMSD) [18], [22]–[24]. The conformations with the minimum MolDock score values are achieved as the optimal docked conformations. In the docking experiments between lignin and ligninolytic enzymes, the most favorable results were for the complexes Lac-lignin, LiP-lignin, and MnP-lignin, showing MolDock score values as -127.77, -156.03, and -142.33, respectively (Table 1). The further accurately docked analysis was performed on the basis of the Re-Rank score function. It is believed that the Re-Ranking score function is generally more reliable than the MolDock score function at selecting the best solution among multiple solutions derived from the same ligand [24]. The method involved was similar to the MolDock score function, except that the Steric (by LJ12-6) terms and torsion term were included [23]. After re-evaluation, the best pose for the complex MnP-lignin examined in the present study exhibited Re-Rank score comparable to that of LiP-lignin or Lac-lignin. In particular, the complexes LiP-lignin and MnP-lignin showed lower scores than the complex Lac-lignin, regardless of whether the standard was based on MolDock score function or Re-Ranking score function.

**Figure 1:**
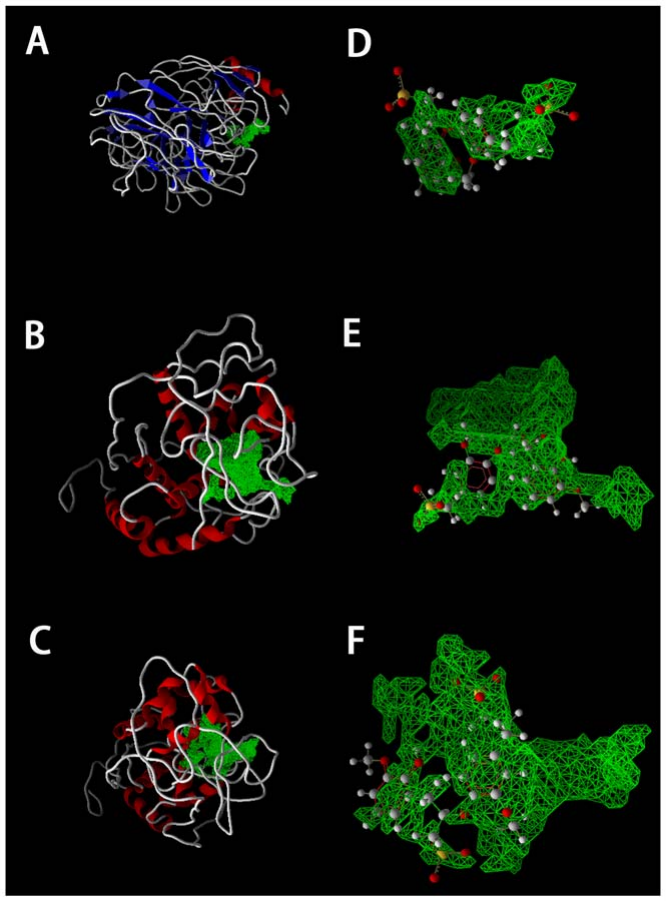
Binding pockets and binding orientations of lignin in the best docking Lac-lignin, LiP-lignin and MnP-lignin complexes. Panels A, B and C display the binding pockets of Lac, LiP and MnP, respectively, whereas panels D, E and F show the binding orientations of Lac, LiP and MnP, respectively. The 3D structures of Lac, LiP and MnP are represented in Cartoon style. The green grids show the binding pockets of lignin-enzymes. The lignin is clearly showed in ball and stick model (colored by element: *gray*, carbon; *red*, oxygen; *white*, hydrogen; *yellow*, sulfur).

The docked orientation for Lac was different from that for LiP or MnP (Fig. 1). In the Lac-lignin complex, lignin located near the surface of the binding pocket, while in the LiP-lignin and MnP-lignin complexes lignin was in the center of the binding pocket and was nearly completely buried within the corresponding binding pocket (Fig. 1). An interesting observation was that LiP-lignin and MnP-lignin with similar binding orientations had very close MolDock/Re-Rank score, whereas Lac-lignin complex having different binding orientation with other two complexes showed higher MolDock/Re-Rank score (Table 1; Fig. 1). This observation suggested that binding orientation was responsible for binding affinity. Previous experiments demonstrated that MnP and Lac could not bind to lignin in a specific manner [14]. The most probable explanation for this observation is that the experimental technologies used in that study could not appropriately identify the direct interactions of MnP and Lac with lignin [14], because later research in which Lac was proved to be capable of oxidising lignin through direct interaction with lignin discredited the previous conclusions by more advanced technologies [13]. It must be noted that the catalytic activity of enzyme cannot be determined by binding affinity or tightness alone [18]. Thus, binding affinity alone is insufficient to explain the observed divergence related to lignin-degrading activity, while other factor such as interaction profile should be considered. Similar MolDock/Re-Rank score and binding orientation between MnP-lignin and LiP-lignin may be derived from the fact that MnP is also heme-containing glycoprotein consistent with LiP [25].

### Interactional analysis and MD simulations

**Figure 2:**
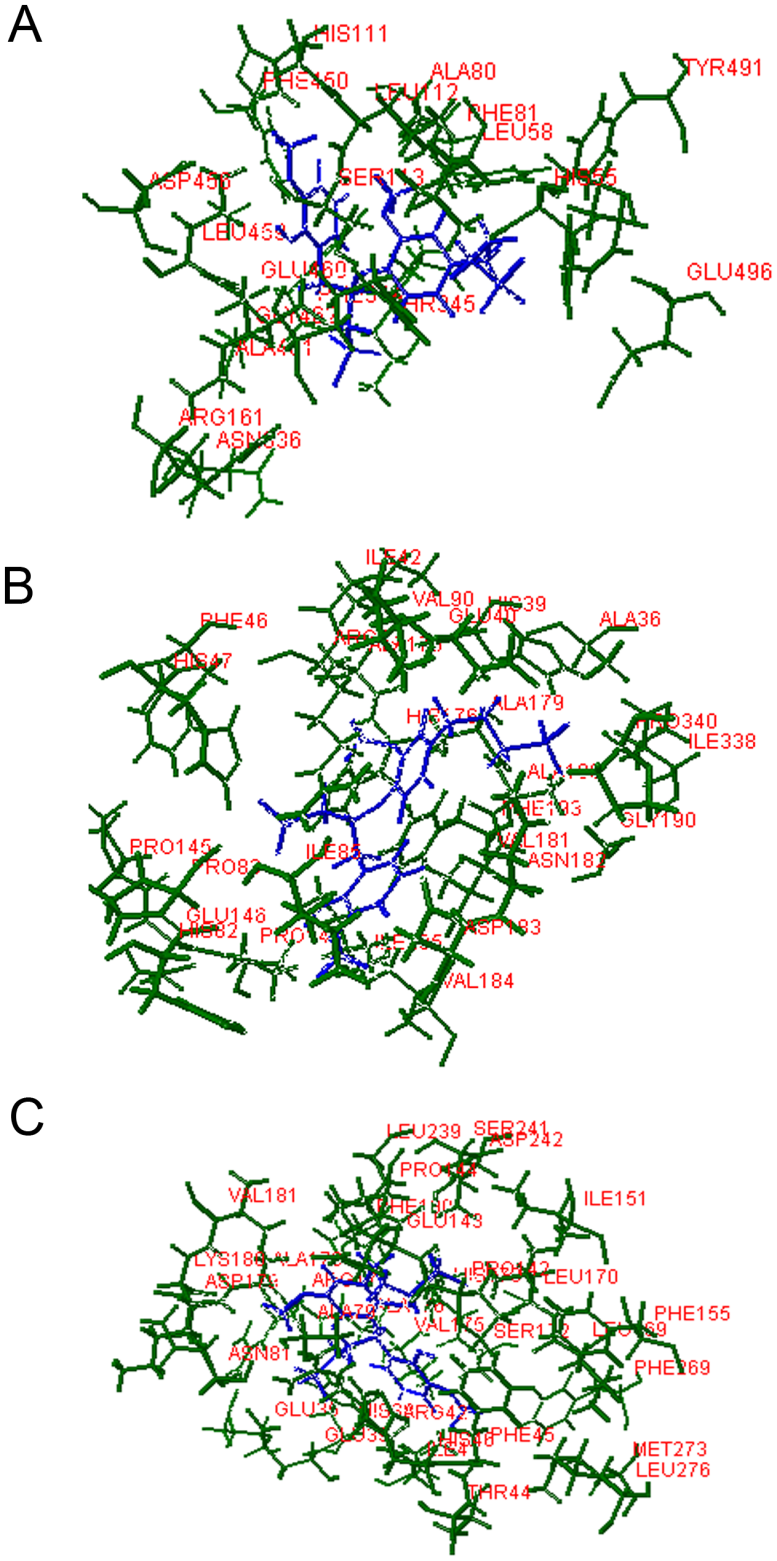
3D stick model representations of binding modes and binding interactions between ligninolytic enzymes and lignin (colored by element: *green*, ligninolytic enzyme; *blue*, lignin). (A) Lac-lignin system. (B) LiP-lignin system. (C) MnP-lignin system.

**Figure 3:**
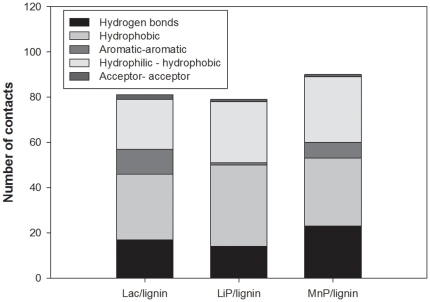
Number of lignin-enzyme contacts in the best docking Lac-lignin, LiP-lignin and MnP-lignin complexes. Analyzed ligand–protein contacts include hydrogen bonding (Hb), hydrophobic (Ph) contact, aromatic-aromatic (Ar) contact, hydrophilic-hydrophobic (HH) contact, and acceptor-acceptor (AA) contact.

The best binding modes of lignin at the three ligninolytic enzymes were shown in Fig. 2. It has been well demonstrated that various ligninolytic enzymes have non-identical ability to degrade lignin [3]. This difference may be partly correlated with their different interactions with lignin [1]. We analyzed the interactions between ligand and receptor residues in the Lac-lignin, LiP-lignin, and MnP-lignin complexes using the LPC/CSU server [26]. The docked conformations of lignin in the active sites of ligninolytic enzymes exhibited similar requirements of molecular contacts: hydrogen bonding (Hb) contact, hydrophobic (Ph) contact, aromatic-aromatic (Ar) contact, hydrophilic-hydrophobic (HH) contact, and acceptor-acceptor (AA) contact were consistently present in each docked complex. Residues GLU460, PRO346 and SER113 in Lac, residues ARG43, ALA180 and ASP183 in LiP and residues ARG42, HIS173 and ARG177 in MnP were most crucial in binding of lignin, respectively (Tables S1, S2 and S3). The Ph contacts for lignin docked into ligninolytic enzymes were shown to be most common among all types of contacts (Fig. 3). LiP was found to have the most abundant Ph contacts with lignin out of all analyzed enzymes. The lignin embeded into MnP formed Ph interactions to the residues ALA176, ALA178, ARG42, ASP179, HIS46, ILE41, LEU176, LEU239, LYS180, PHE190, PHE45, PRO142, PRO144, VAL175 and VAL181 (Table 2). The docking conformation for Lac-lignin complex indicated that the lignin had the least Ph contacts with the residues ALA80, ARG157, GLN499, GLU460, LEU112, LEU459, LEU58, PHE81, PRO346 and SER113 among all surveyed ligninolytic enzymes (Table 2). Lac, LiP and MnP, however, revealed a close HH and comparable AA interactions with lignin (Fig. 3). The most common HH contacts for ligninolytic enzymes as revealed in the present study were observed in MnP-lignin. LPC/CSU calculation showed seven and two more HH contacts for MnP than Lac and LiP, respectively. Some HH interaction residues were the same in Lac-lignin, LiP-lignin, and MnP-lignin complexes: PRO and ARG. Among the AA interactions, one residue GLU occurred in each of Lac-lignin and LiP-lignin. For complex MnP-lignin, lignin had AA contact with residue ARG177 which is able to restrict the movement of Mn^II^ ligand GLU35 [27]. The selected lignin in Lac formed Hb contacts to ARG157, ARG161, ASN336, GLN499, GLU460, GLU496, GLY462, HIS55, PHE344 and SER113 in addition to Ar interactions with PHE81, PHE344 and PHE450. In the LiP-lignin complex, lignin generated Hb contacts with residues ALA180, ARG43, ASN182, GLU40, HIS176, HIS39, HIS47, ILE338, PRO145 and PRO83 with Ar interaction with PHE193. The whole part of lignin structure was inserted deeply in the binding pocket of MnP, forming Hb contacts to the residues ARG177, ARG42, ASP179, ASP241, ASP242, GLU143, GLU35, GLU39, HIS173, HIS38, HIS46, SER172 and SER241. Interestingly, GLU35, GLU39 and ASP179 were also found to be involved in the binding of Mn^II^ in crystal structure of MnP [25], [28]. ARG42 was the most abundant residue forming contacts with lignin in MnP-lignin complex (Table S2). This can be expected, since ARG42 is very important for peroxidase function [29]. Ar contacts were produced by the residues PHE and HIS in the MnP-lignin complex. An important finding was that PHE was a common residue forming the Ar contacts with lignin in all analyzed enzymes. In relation to hydrophobic interactions, three types of amino acid residues ALA, ARG, and PRO were observed in each complex. The interaction profile for LiP-lignin in this study differed from that of the previous study in which His…Asp…proximal-His motif from LiP was proposed to be responsible for lignin oxidation [14]. This difference may be because these two studies selected different lignin.

**Table 2:** Interactional residues of ligninolytic enzymes with lignin.

Enzyme-lignin complex^a^	Hb	Ph	Ar	HH	AA
Lac-lignin	ARG157, ARG161, ASN336, GLN499, GLU460, GLU496, GLY462, HIS55, PHE344, SER113	ALA80, ARG157, GLN499, GLU460, LEU112, LEU459, LEU58, PHE81, PRO346, SER113	PHE81, PHE344, PHE450	ARG157, GLN499, GLU460, LEU459, LEU58, PHE344, PRO346, THR345, TYR491	GLU460, SER113
LiP-lignin	ALA180, ARG43, ASN182, GLU40, HIS176, HIS39, HIS47, ILE338, PRO145, PRO83	ALA175, ALA179, ALA180, ARG43, ASP183, GLU40, HIS176, HIS39, ILE235, ILE338, ILE42, ILE85, PHE193, PRO147, VAL181, VAL184, VAL90	PHE193	ALA179, ALA180, ALA36, ARG43, ASN182, HIS176, HIS39, ILE235, ILE338, ILE85, PRO147, PRO340, VAL184	GLU146
MnP-lignin	ARG177, ARG42, ASP179, ASP241, ASP242, GLU143, GLU35, GLU39, HIS173, HIS38, HIS46, SER172, SER241	ALA176, ALA178, ARG42, ASP179, HIS46, ILE41, LEU176, LEU239, LYS180, PHE190, PHE45, PRO142, PRO144, VAL175, VAL181	HIS173, PHE190	ALA178, ALA79, ARG127, ARG42, GLU39, HIS173, HIS38, HIS46, ILE151, LEU170, LEU239, PHE45, PRO142, PRO144, VAL181	ARG177

a Refers to the best docking complex.

LPC/CSU server was used to analyze ligand–protein contacts, including hydrogen bonding (Hb) contact, hydrophobic (Ph) contact, aromatic-aromatic (Ar) contact, hydrophilic-hydrophobic (HH) contact, and acceptor-acceptor (AA) contact.

Overall, the interaction profile for LiP-lignin was different from that for Lac-lignin, but relatively similar to that for MnP-lignin. This similarity and difference may be once again attributed to the nature of their binding orientations. Analysis of binding models for several alkyl phenols polymerization could be applied in the design of new CIP variants to achieve better polymerization activity [17]. Similarly, our docking experiments could be also used to design promising ligninolytic enzyme mutants with better lignin-degrading activity.

**Figure 4:**
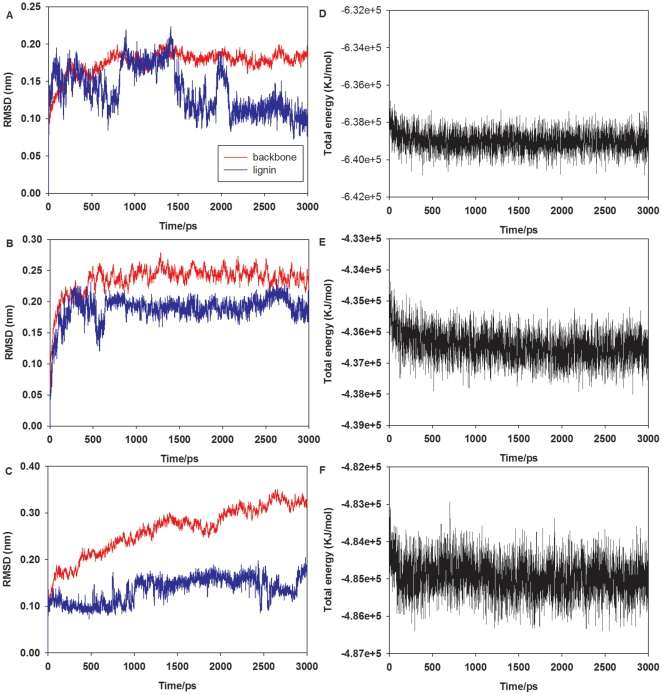
RMSD obtained during 3000 ps MD simulations for the backbones (red lines) and lignin (blue lines) from the corresponding starting structures of Lac-lignin (A), LiP-lignin (B) and MnP-lignin (C) complexes as a function of the simulation time, and plots of total energy vs. simulation time (D, Lac-lignin system; E, LiP-lignin system; and F, MnP-lignin system).

The robustness and stability of the predicted 3D structures of Lac-lignin, LiP-lignin and MnP-lignin complexes were further determined and verified by MD simulations. According to the 3000 ps MD simulation for the structure of Lac-lignin complex, the RMSD for the backbone of Lac as a function of the simulation time became stable (Fig. 4A). Clearly, this protein backbone quickly became equilibrated after 500 ps with a mean RMSD value of 1.5 Å for the backbone but the lignin did not become stable until after 2100 ps. Figure 4B showed the LiP-lignin system did not equilibrate in the first 800 ps and then was relatively stable in the following 2200 ps; the average RMSD value was 2.4 Å for backbone of LiP and 2.0 Å for lignin, respectively. From Fig. 4C, MnP-lignin with respect to the corresponding starting structure was also stable after 1000 ps, with a mean RMSD value of 2.4 Å for backbone and 1.4 Å for the lignin. The total energy for each complex was analyzed, being found to be stable throughout the simulation process (Figs. 4D, E and F). Low backbone and ligand RMSD values as well as stable total energy confirmed the stability of Lac-lignin, LiP-lignin and MnP-lignin systems and the credibility of the docking results [15]. Noteworthy, other enzymes (are not main enzymes for lignin decay) such as mycelium-associated dehydrogenases, oxidases generating H_2_O_2_, aryl-alcohol dehydrogenases (AAD) and quinone reductases (QR) are also associated with lignin biodegradation [3]. However, their 3D structures are rarely reported in PDB. Their interaction mechanisms with lignin merits further investigation.

### Conclusion

We have successfully identified the binding modes of ligninolytic enzymes to lignin. Analyses of binding orientations and interactions between lignin and ligninolytic enzymes are possible to be helpful in understanding their lignin-degrading mechanisms, because the direct interactions have been found to be correlated with lignin biodegradation. Our study provides the basis to design more selective and potent ligninolytic enzyme mutants for lignin biodegradation. To our best knowledge, this is a first analysis of the interactions between lignin and its degradation enzymes for such purposes and represents a general method for study of other interactions from various biodegradation processes or pollutant treatments.

## Materials and Methods

The crystal structures of Lac, LiP and MnP were downloaded from the Protein Data Bank (PDB) (http://www.pdb.org/pdb/home/home.do) [30]. Their PDB IDs and resolution (*R*) are 1GYC (*R = 1.90 Å*) [7], 1LLP (*R = 1.70 Å*) [6] and 3M5Q (*R = 0.93 Å*) [2], respectively. The bound ligands of each analyzed enzyme were deleted. The chemical 2D structure of lignin derivative in SDF format with ID 167333 was obtained from PubChem (http://pubchem.ncbi.nlm.nih.gov/) [31], and was used as a lignin model substrate for exploring the interactions of ligninolytic enzymes with lignin. Its 3D conformation was further generated and optimized as docking ligand.

MVD, a graphical-automatic docking software, was utilized to perform docking of lignin into the binding pockets of ligninolytic enzymes [23]. This tool has been reported to have high accuracy and versatility [24]. Each enzyme was analyzed separately. The bond order and the atom types of ligninolytic enzymes and lignin structures were automatically corrected with the correct charges assigned during the preparation process. Potential binding pockets (also named cavities or active sites) were detected by use of the cavity detection algorithm of MVD. Docking was performed using the MolDock scoring function (MolDock Score) together with the Moldock SE algorithm. This algorithm applied a maximum population size of 50 individuals. Maximum interactions, number of runs, energy threshold, maximum steps, and neighbour distance factor were set to 1500, 10, 100.00, 300, and 1.00, respectively. The best conformations with the lowest docked energy were chosen from all generated conformations. For each best conformation, we used the LPC/CSU server to analyze ligand–protein contacts, including hydrogen bonding (Hb) contact, hydrophobic (Ph) contact, aromatic-aromatic (Ar) contact, hydrophilic-hydrophobic (HH) contact, and acceptor-acceptor (AA) contact [26].

MD simulations for the obtained complexes of lignin with Lac, LiP and MnP were performed using the standard GROMOS96 force field, implemented in GROMACS 4.0.7 software package [32]–[33]. The topology file was built using PRODRG program [34]. We neutralized the charges of each complex with Na^+^ ions. The SPC216 water model was used for the solvation of all complexes. The Particle Mech Ewald (PME) method was applied to the treatment of long-range electrostatic interactions [35]. A steepest descents minimization was used to release bad van der Waals contacts. Subsequently, three 3000 ps MD simulations at 300 K and 1 bar pressure were carried out.

## Supporting Information

Table S1 List of residues forming contacts with the ligand lignin in PDB entry 1LLP.
(DOC)

Table S2 List of residues forming contacts with the ligand lignin in PDB entry 3M5Q.
(DOC)

Table S3 List of residues forming contacts with the ligand lignin in PDB entry 1GYC.
(DOC)
