# Supplementary material for: Understanding Lignin-Degrading Reactions of Ligninolytic Enzymes: Binding Affinity and Interactional Profile
Source: PLoS One. 2011 Sep 29;6(9):e25647. doi: 10.1371/journal.pone.0025647 (PMC3183068; doi:10.1371/journal.pone.0025647)
Supplement: Table S1 — List of residues forming contacts with the ligand lignin in PDB entry 1LLP. (DOC) [file pone.0025647.s001.doc]

**Table S1. List of residues forming contacts with the ligand lignin in PDB entry 1LLP**

| Residue | Number of contacts |
| --- | --- |
| ALA36 | 2 |
| HIS39 | 8 |
| GLU40 | 5 |
| ILE42 | 1 |
| ARG43 | 22 |
| PHE46 | 2 |
| HIS47 | 6 |
| HIS82 | 2 |
| PRO83 | 1 |
| ILE85 | 5 |
| VAL90 | 1 |
| PRO145 | 3 |
| GLU146 | 3 |
| PRO147 | 9 |
| ALA175 | 3 |
| HIS176 | 9 |
| ALA179 | 3 |
| ALA180 | 11 |
| VAL181 | 5 |
| ASN182 | 7 |
| ASP183 | 10 |
| VAL184 | 8 |
| GLY190 | 1 |
| PHE193 | 3 |
| ILE235 | 3 |
| ILE338 | 6 |
| PRO340 | 1 |
