# Supplementary material for: Understanding Lignin-Degrading Reactions of Ligninolytic Enzymes: Binding Affinity and Interactional Profile
Source: PLoS One. 2011 Sep 29;6(9):e25647. doi: 10.1371/journal.pone.0025647 (PMC3183068; doi:10.1371/journal.pone.0025647)
Supplement: Table S2 — List of residues forming contacts with the ligand lignin in PDB entry 3M5Q. (DOC) [file pone.0025647.s002.doc]

**Table S2. List of residues forming contacts with the ligand lignin in PDB entry 3M5Q**

| Residue | Number of contacts |
| --- | --- |
| GLU35 | 2 |
| HIS38 | 7 |
| GLU39 | 7 |
| ILE41 | 5 |
| ARG42 | 20 |
| THR44 | 1 |
| PHE45 | 9 |
| HIS46 | 5 |
| ALA79 | 1 |
| ASN81 | 1 |
| PRO142 | 8 |
| GLU143 | 2 |
| PRO144 | 5 |
| ILE151 | 3 |
| PHE155 | 2 |
| LEU169 | 1 |
| LEU170 | 3 |
| SER172 | 7 |
| HIS173 | 13 |
| VAL175 | 3 |
| ALA176 | 5 |
| ARG177 | 11 |
| ALA178 | 3 |
| ASP179 | 4 |
| LYS180 | 3 |
| VAL181 | 4 |
| PHE190 | 5 |
| LEU239 | 5 |
| SER241 | 6 |
| ASP242 | 1 |
| PHE269 | 2 |
| MET273 | 1 |
| LEU276 | 1 |
