# Supplementary material for: Understanding Lignin-Degrading Reactions of Ligninolytic Enzymes: Binding Affinity and Interactional Profile
Source: PLoS One. 2011 Sep 29;6(9):e25647. doi: 10.1371/journal.pone.0025647 (PMC3183068; doi:10.1371/journal.pone.0025647)
Supplement: Table S3 — List of residues forming contacts with the ligand lignin in PDB entry 1GYC. (DOC) [file pone.0025647.s003.doc]

**Table S3.** **List of residues forming contacts with the ligand lignin in PDB entry 1GYC**

| Residue | Number of contacts |
| --- | --- |
| HIS55 | 4 |
| LEU58 | 7 |
| ALA80 | 5 |
| PHE81 | 6 |
| HIS111 | 6 |
| LEU112 | 5 |
| SER113 | 13 |
| ARG157 | 8 |
| ARG161 | 2 |
| ASN336 | 1 |
| PHE344 | 6 |
| THR345 | 6 |
| PRO346 | 13 |
| PHE450 | 5 |
| ASP456 | 1 |
| LEU459 | 7 |
| GLU460 | 15 |
| ALA461 | 1 |
| GLY462 | 1 |
| TYR491 | 2 |
| GLU496 | 4 |
| GLN499 | 6 |
